# Reform in the West African Medical Service

**Published:** 1909-07-17

**Authors:** 


### The Hospital

A Journal of

The Medical Sciences and Hospital Administration.

New Series. No. 125, Vol. V. [No. 1197, Vol. XLVI.] SATURDAY, JULY 17, 1909.

### REFORM IN THE WEST AFRICAN MEDICAL SERVICE.

The Departmental Committee's report on the organisation of the West African Medical Service has just been issued, and it contains material for careful consideration on the part of medical school authorities and recently qualified medical men. In the first place, and by way of introduction to their formal recommendations, the committee express the opinion, based upon the evidence which they have received, that medical service in West Africa deserves to be considered by junior medical practitioners as a promising and permanent career. From certain documents submitted to them, and, we may perhaps add, by common consent amongst those who are in touch with the subject, the committee feel quite certain that a different view has hitherto been taken. In accordance with this prevalent idea, young medical practitioners have so far been induced to join the service for a limited period of time only, with the implied suggestion that it would be best for them to leave West Africa before it was too late to set up in practice in England; thus, their retirement, with a gratuity of £1,000 at the end of nine years' service, has been officially, or at least semi-officially, regarded as being in the best interests both of the public service and of the medical officers themselves. The committee dissent from this view. They understand that, after nine years' West African service, a medical officer is not well suited for general practice in the United Kingdom. Moreover, it seems clear that the majority of medical officers already in this service would much prefer not to begin a new mode of professional life after eight or nine years under Government employment, but to make the Colonial Medical Service their career. Further, the committee are strongly of opinion that it is not in the best interests of the West African Colonies and Protectorates that medical men of experience should be removed from the Colonial Service after so short a period as nine years, of which some six years only have actually been spent in West Africa. They consider—and, general medical opinion will surely agree with them—that the continued presence of medical men trained in the study of tropical disease, and fully acquainted with local conditions, is of immense importance, especially for the elucidation of the problems of preventive medicine in West Africa. They point out that the lack of continuity in administration, acknowledged to be the chief defect of tropical African Government, will tend to increase if experienced officers are encouraged to retire early.

The committee thus base their report upon the view that medical officers should be encouraged to make the West African service their permanent career. Promotion is at present very slow; and officers may remain for years at a salary of £500 a year. The committee are convinced that the slowness of promotion and meagreness of remuneration which exists throughout the West African medical service, are mainly responsible for the discontent in the staff. They recommend that the rates of pay should be improved. A still more important proposal is to the effect that a higher and better-paid rank in the West African medical service should be instituted. This will afford opportunities for the advancement of officers of merit and enthusiasm, who up till now have been discouraged through the operation of a grotesque official misconception. The dangers to health and life said to be incident to a career in this region of the tropics seem to have been magnified unduly into an excuse for prejudicing the welfare and progress of the community, as well as of its individual medical officers. It may have been true a few years ago that after nine years' service few civil officers of the administration were likely to render valuable service by continued residence: and this may have been especially true of the medical officers, the nature of whose duties required their residence in the districts where sickness, particularly malaria, was most prevalent and most malignant. At that time, say a score of years ago, it is probable that the percentage of those who survived a stay of eighteen years on "the Coast," and lived to enjoy the full pension that could thus be earned, was very small. But the new way of life which has come to be the rule instead of the exception in West Africa has changed all this. Temperance is now the rule instead of the rare exception: sanitation is attended to instead of totally neglected: malaria and other tropical diseases have been shorn of much of their terror: everything, in a word, that is comprised within the science of tropical hygiene has made such strides that the West Coast is no longer pre-eminently the white man's grave.

The natural consequence is that, after nine years' service, officials are no longer cachectic and fever-stricken shadows, but men in the full tide of vigour and of a ripe experience in their duties: another consequence is that the number of those who retire after eighteen years' service to spend many years of healthy life on very comfortable pensions is steadily increasing. These recommendations will, if carried into effect, both attract a better class of candidate in the future, and ameliorate the lot of those who are already members of the West African Medical Service.

